# The nexus between sleep disturbances and mental health outcomes in military staff: a systematic review

**DOI:** 10.5935/1984-0063.20220063

**Published:** 2022

**Authors:** Negin Farhadian, Alireza Moradi, Mohammad Nami, Kamran Kazemi, Mohammad Rasoul Ghadami, Alireza Ahmadi, Reza Mohammadi, Mohammad Naseh Talebi, Prasun Chakrabarti, Babak Kateb, Habibolah Khazaie

**Affiliations:** 1 Kermanshah University of Medical Sciences, Sleep Disorders Research Center, Health Institute, Kermanshah, Iran; 2 Kharazmi University, Department of Clinical Psychology - Tehran - Iran; 3 Shiraz University of Medical Sciences, Department of Neuroscience - Shiraz - Iran; 4 Shiraz University of Technology, Department of Electrical and Electronics Engineering - Shiraz - Iran; 5 Kermanshah University of Medical Sciences, Department of Anesthesiology - Kermanshah - Iran; 6 Karolinska Institute, Department of Caring Science and Society - Huddinge - Sweden; 7 Institute for Cognitive Science Studies, Cognitive Science Studies - Tehran - Iran; 8 Techno India NJR Institute of Technology, Technology - Rajasthan - India; 9 National Center for NanoBioElectoronics, Brain Technology and Innovation Park - Los Angeles - United States

**Keywords:** Depression, Anxiety, Stress Disorders, Post-Traumatic, Military Personnel, Sleep

## Abstract

**Objectives:**

Military personnel are unique occupational groups who happen to frequently experience sleep insuffciencies. Since sleep disorders are known to be linked to many psychiatric symptoms, sleep disturbance is a salient concern among active duty service members and veterans. Existing evidence indicates that although sleep disturbances co-occur with mental illnesses, there is a tendency to particularly label them as consequences of certain mental health issues.

**Material and Methods:**

This review focuses on the emerging evidence which identifies sleep disturbances as a precursor for mental illnesses. In this regard, the impact of sleep disturbance on the development of mental health outcomes including post-traumatic stress disorder (PTSD), depression, and anxiety has been thoroughly scrutinized. A systematic search was conducted using PubMed, Scopus, and Web of Science academic databases using appropriate keywords.

**Results:**

Reviewed evidence substantiates the predicting role of sleep complaints and disorders to herald PTSD, depression, and anxiety among military staff.

**Conclusion:**

Early diagnosis of sleep disturbances and properly addressing them in active-duty service members and veterans should be then sought to prevent the development and progression of consequent mental health- related comorbidities in this study group.

## INTRODUCTION

Sleep plays a central role in maintaining psychological and physical well-being in military staff and veterans. The close link between sleep disturbances and mental health disorders is a key concern among military members as they face several environmental stressors and challenges. This has potentially exposed military staff at an increased risk for sleep predicaments compared to civilians^1^. They are several factors to justify sleep disturbances among military staff from which exposure to combat stress^2^-^5^, frequent shift work, frequent changes in duty assignments, and changes in duty station are more significant^6^,^7^. Inadequate sleep in military personnel is shown to hinder their operational readiness, effectiveness, and safety through impaired attention/concentration, judgment, responsiveness, and decision making as key substrates of their cognitive performance^8^,^9^. On the other hand, insufficient sleep corresponds to an increased risk of cardiovascular disease, obesity, and diabetes^10^,^11^. Moreover, there is a compelling body of evidence suggesting that insufficient sleep results in diminished pain threshold, post-traumatic stress disorder (PTSD), depression, anxiety, and robust symptoms following a traumatic brain injury (TBI)^12^. Aggravation of such mental-health disorders are in turn associated with chronic medical and often psycho-somatic conditions such as cardiovascular diseases, hypertension, and asthma^13^,^14^.

Given the above, both sleep predicaments and mental health disorders are known to team-up with impaired functionality, increased healthcare utilization, as well as increased health-related costs and reduced quality of life^15^-^18^. Based on a recent investigation, sleep problems over post-deployment in the Afghanistan war were related to the probable mental illness symptoms^19^,^20^. Short sleep among redeployed operation Iraqi freedom soldiers were significantly associated with a dramatic increase in symptoms of PTSD, depression, panic syndrome, and high-risk behaviors such as alcohol, tobacco misuse and suicidal attempts^21^. The high rate of comorbid sleep disturbances along with mental disorders among active duty service members and veterans signifies the necessity for continued focused research on this specific topic.

The present systematic review has been an attempt to evaluate the association between sleep disturbances and mental health outcomes in active-duty service members and veterans. The term ‘sleep disorders’ represents short and insufficient sleep, poor sleep quality, insomnia, sleep apnea and nightmares. Studies have ubiquitously indicated that sleep disturbance is a notable risk factor, which predisposes military staff to specific mental disorders including PTSD, depression, and anxiety-related symptoms.

## MATERIAL AND METHODS

The present systematic review included studies that examined the association of sleep disturbance with mental disorders including PTSD, depression, and anxiety in active duty service members and veterans. This study was conducted in accordance with the recommendations laid down by the PRISMA (Preferred Reporting Items for Systematic Reviews and Meta-Analyses) guideline. The approach comprised a 27-item checklist and a four-phase flow diagram to retrieve selected documents.

Systematic information retrieval was performed in Scopus, PubMed and Web of Science academic databases. Search terms were combined using the Boolean operators ‘and’ between categories and ‘or’ within categories (Table 1). For PubMed, MeSH (Medical Subject Headings) terms were also used.

**Table 1:** Search strategy (Boolean operators ‘or’ and ‘and’ between columns).

Sleep disturbances	Mental disorder	Military personnel
Sleep disturbances, sleep disorder, sleep difficulties	Mental disorder, mental health	Military personnel, army personnel, armed forces personnel

Notes: CAPS = Clinician administered PTSD scale, DSM-IV Version; PHQ = Patient health questionnaire; PCL-C = PTSD Checklist, civilian version; SCIDI/P = Structured clinical interview for DSMIV-TR axis I disorders; PSQI-A = Pittsburgh sleep quality index-addendum; PCL-M = PTSD checklist, military version; BDI-II = Beck depression inventory-II; PSG = Polysomnography; CIDI-SC = Composite international diagnostic interview screening scales; SRIP = Self-rating inventory for PTSD; SCL-90 = Dutch version of the symptom checklist; PDHA = Post-deployment health assessment; PDHRA = Post-deployment health reassessment; PC-PTSD = Primary care PTSD screen; GAD-7 = Generalized anxiety disorder screener; GSI = Global symptom index; SCL = Symptom Checklist-90-Revised; K10 = Kessler psychological distress scale 10; HADS = Hospital anxiety and depression scale; ICD-9 = International classification of diseases; DTS = Davidson trauma scale; SAS = Self-rating anxiety scale; DSI-SS = Depressive symptom inventory suicidality subscale.

Exclusion of an article were based on the following criteria: (i) studies focusing only on the mental health status or sleep disturbances among military members; (ii) studies that examined the effects of any stimulation or pharmacological and psychological interventions on sleep complaints and mental disorders among military members.

Furthermore, investigations focusing on the impact of sleep disturbances on specific mental disorders including PTSD, depression and anxiety were included, while those addressing mental health risk behaviors like suicide, substance and alcohol abuse were disregarded.

## RESULTS

**Figure 1:**
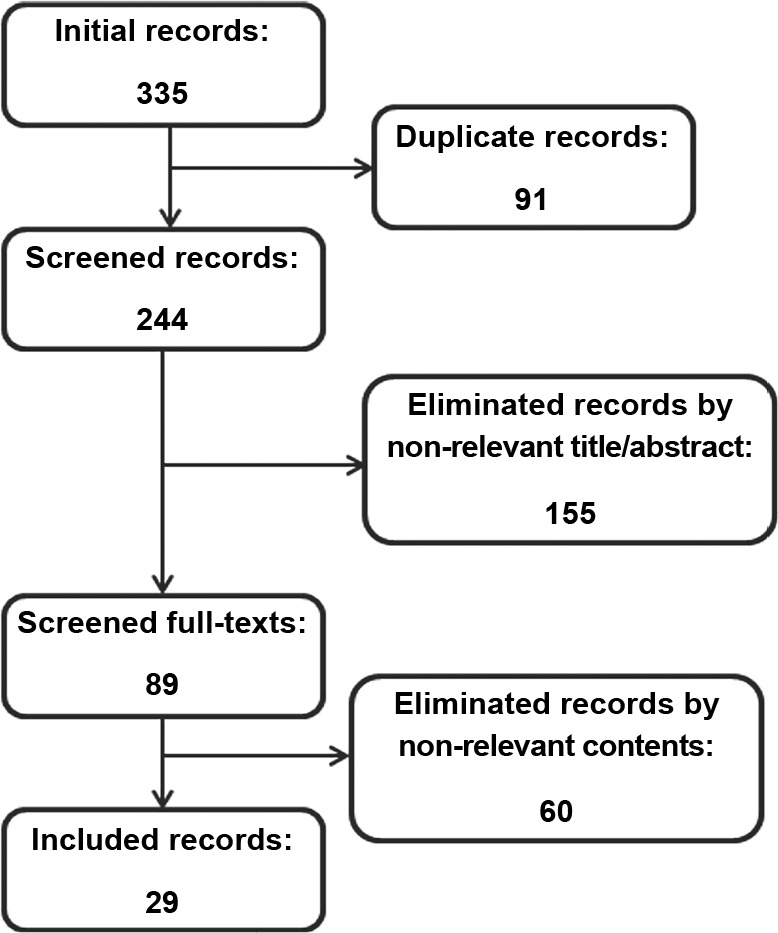
Diagram of systematic search strategy and retrieval of the records.

From a total of 335 publications retrieved, 91 duplicates, 17 reviews, and 198 non-relevant records were excluded. Subsequently, 29 studies met the inclusion criteria (Figure 1). In addition, from the 29 isolated studies, a total of 12 documents were examining the relationship between sleep disturbances and PTSD symptoms, 4 studies were describing the prediction role of sleep in context to the combination of PTSD, depression and anxiety symptoms, while 3 records assessed the effect of sleep complaints and resultant depression. Between the lines, 7 studies evaluated the association between sleep disturbances and mental health disorders. Few records were found to be exclusively devoted to evaluating the effect of sleep disorders on the development of depression and anxiety symptoms. Summary of the reviewed studies is represented in Table 2. Additionally, main findings regarding the association between sleep complaints and mental health disorders, specifically PTSD, depression, and anxiety symptoms are articulated in the present review.

**Table 2:** Summary of the enrolled studies.

Study	Type of study	No of participants	Population	Method of sleep disorder assessment	Method of mental disorder assessment
Acheson et al. (2019)^1^	Cohort	2,404	Marines and Navy Corpsmen deployed to Iraq and Afghanistan	Sleep disturbances by BDI insomnia; CAPS average hours of sleep; CAPS difficulty falling or staying asleep and 6 items from the CAPS serving as indicators of the construct ‘re-experiencing symptoms’	CAPS
Gehrman et al. (2013)^22^	Cohort	15,204	Active service	Sleep item from the PCL-C and PHQ for insomnia symptoms	17-item PCL-C and PHQ-9
Swinkels et al. (2013)^23^	Cross-sectional	1,640	U.S. Afghanistan/Iraq era veterans	PSQI-A	SCIDI/P
Koffel et al. (2013)^24^	Longitudinal	522	U.S. National guard soldiers deployed to Iraq	Sleep-related items within the BDI-II referring to loss of energy	CAPS, PCL-C at pre-deployment and the PCL-M at post-deployment
Taylor et al. (2014)^25^	Cross-sectional	3,175	U.S. active duty and reserve Navy personnel	Self-reported sleep measures	DSM-IV and PHQ
McLay et al. (2010)^26^	Retrospective	1,887	U.S. military	PCL-M	PCL-M
Wright et al. (2011)^27^	Longitudinal	659	Active duty soldiers	Insomnia severity index	PHQ, PCL,
Mysliwiec et al. (2013)^28^	Cross-sectional	110	U.S. military personnel	PSG	PCL-M
Taylor et al. (2016)^7^	Cross-sectional	4,101	Active duty service members	Insomnia severity index	PCL-M
Brownlow et al. (2017)^29^	Cross-sectional	29,674	Military soldiers	Brief insomnia questionnaire	CIDI-SC, PCL
Wang et al. (2019)^30^	Prospective	4645	U.S. Army soldiers	Brief insomnia questionnaire	PCL and CIDI-SC
Hansen et al. (2018)^31^	Cohort	438	Army national guard soldiers	Insomnia severity index	The primary care-post-traumatic stress disorder screen
Liempt et al. (2013)^33^	Prospective cohort	453	NATO-International security assistance force	SRIP, SCL-90	SRIP
Steele et al. (2017)^34^	Cross-sectional	972	U.S. Navy sailors and marines	Self-report survey	PCL, PHQ
Macera et al. (2013)^35^	Prospective	29,640	U.S. Navy and Marine Corps	PDHA	PDHA, PDHRA, PC-PTSD
Osgood et al. (2019)^36^	Cross-sectional	2420	Soldiers	Self-report survey	PCL-C, PHQ-8, GAD-7
Lewis et al. (2009)^37^	Pilot	152	Australian Vietnam war veterans	PSQI	PCL
Hughes et al. (2018)^38^	Cross-sectional	1,118	U.S. military veterans	PSQI, PSQI-A	GSI, SCL
Hunt et al. (2016)^39^	Prospective	1636	U.K. Armed forces	PCL-C, GHQ-12	PCL-C, GHQ-12
Kim et al. (2016)^40^	Cross-sectional	5,764	Korea armed forces	Self-reported questionnaire	K10
Hougsnæs et al. (2017)^41^	Retrospective	3403	Norwegian soldiers	Insomnia severity index	PCL-M-17, HADS
Seelig et a. (2010)^20^	Longitudinal cohort	41,225	Service members, reserve/guard personnel, veterans	PHQ, PCL-C	PCL-C
Soreca et al. (2019)^42^	Cross-sectional	33,818	U.S. military veterans	Breathing-related sleep disorder of ICD-9	ICD-9
Ulmer et al. (2015)^43^	Cross-sectional	1,238	Veterans and active duty military personnel	PSQI, SCL-90, DTS	SCID-I
Wang et al. (2020)^44^	Cross-sectional	489	Military officers and soldiers	PSQI	SAS
Chou et al. (2016)^45^	Cross-sectional	720	Taiwanese army, air force, marines and military police	PSQI	BDI
Tonon et al. (2020)^46^	Cross-sectional	236	Male recruits in compulsory military service	PSQI	BDI
Hom et al. (2016)^47^	Prospective	2596	U.S. Army recruiters	Insomnia severity index	DSI-SS

### Post-traumatic stress disorder

In a prospective longitudinal cohort of 2,404 Marines and Navy corpsmen, Acheson et al. (2019)^1^ reported a significant relationship between sleep disturbances on the pre-deployment and latent PTSD symptomology. Additionally, a combat-stress severity showed a small arbitrary effect for such correlations^1^. Pre-deployment short sleep duration (<6h) and insomnia significantly contributed to the developing new-onset PTSD symptoms in a large, population-based cohort study. Also, increased odds (approximately 2 times) of developing PTSD were observed in the military personnel who reported combat-related trauma^22^.

Likewise, in a sample of 1,640 U.S. Afghanistan/Iraq era veterans, 49% endorsed sleeping <6h, 23% slept between 6-7h, 25% slept >7 but <9h, and only 3% slept ≥9h per night. According to the related report, 72% of the sample had poor sleep quality. Specifically, short (≤5h) or long (≥9h) sleep duration and poor sleep quality in this population were attributed to increased odds of PTSD^23^. Interestingly, the preexisting daytime and nighttime sleep complaints were predicting PTSD particularly at futuristic time points up to 2 years after deployment^24^.

Based on another report, among 3,175 active duty and reserve U.S. Navy personnel deployed to a combat zone, two-thirds declared an average of less than 6h sleep per 24h and hence, were classified as sleep deficient. Adjusted for covariates, sleep deficit was the main risk factor for developing PTSD (OR=10.7; 95%CI: 4.3-26.8)^25^.

Similarly, findings from a retrospective cohort of 1,887 U.S. military personnel demonstrated insomnia as the most common PTSD-provoking symptom for those returned from deployments. In fact, while various intensities of insomnia symptoms were observed in 33% of this population, insomnia was more severe in PTSD sufferers upon follow-up^26^. Furthermore, a longitudinal assessment of the association between insomnia and psychological symptoms like PTSD and depression among combat veterans of the Iraq war demonstrated that insomnia at an earlier time-point was a more-likely predictor for PTSD symptoms rather than a later time-point^27^. In a cross-sectional study of 110 active-duty military personnel who returned from deployment, 88.2% of subjects were diagnosed with sleep disorders. Notably, among this population, 25.5% and 24.5% of them fulfilled the diagnostic criteria for insomnia and OSAHS (obstructive sleep apnea-hypopnea syndrome), respectively. Further to this, 38.2% were reported to have comorbid OSAHS and insomnia. Military staff with comorbid OSAHS and insomnia showed more symptoms compatible with PTSD compared to the control subjects without any sleep disorder and those with OSAHS alone^28^.

The prevalence of insomnia among 4,101 active-duty service members was 19.9%. The rates of clinically significant PTSD in the U.S. Army personnel with insomnia were higher (55.5%) than the non-insomniacs (13.5%). Reciprocally, individuals with clinically significant PTSD were more likely to report insomnia (OR=2.39, 95%CI: 1.88-3.04)^7^.

U.S. military soldiers with current PTSD had a high prevalence of insomnia complaints (69.7%) where PTSD symptoms moderated the relationship between insomnia and memory and concentration problems^29^. Adjusting for prior deployment history and sociodemographic characteristics, pre-deployment insomnia among U.S. Army soldiers was related to the increased odds of PTSD following deployment (AOR: 3.14, 95%CI: 2.58-3.82)^30^.

Among soldiers with a rare history of these problems at baseline, pre-deployment insomnia accounted for a 55% increased risk of incident PTSD^30^. In addition, amongst 438 army soldiers of the Minnesota National Guard, a prevalence of moderate to severe levels of insomnia was reported 16.4% and 18.1%, respectively, in those who were primarily screened positive for PTSD. In this study, PTSD was an independent predictor for sleep problems as assessed by the insomniacs^31^. Interestingly, unlike insomnia, nightmare disorder before deployment, predicted PTSD symptoms post-deployment.

Adding to the above, in a sample of 453 military personnel deployed to Afghanistan, a correlation was observed between the number of OSAHS and PTSD severity for those with PTSD. Also, according to polysomnographic registrations and blood samples, increased sleep fragmentation, frequent nightmares, and decreased growth hormone secretion were observed in PTSD patients^32^.

Moreover, a prospective longitudinal cohort including 453 subjects demonstrated an increased risk for the development of PTSD symptoms as a result of pre-deployment nightmares. Nightmares prior to deployment predicted PTSD (OR=2.992; 95%CI: 1.096-8.551), whereas insomnia complaints before deployment did not predict the same (OR=0.976; 95%CI: 0.862-1.155)^33^.

Steele et al. (2017)^34^ examined the impact of sleep problems such as nightmares, difficulty falling asleep, concern resulting from lack of sleep and difficulty staying asleep on the link between combat experiences, and PTSD symptoms in a population of 972 U.S. Navy sailors. Among such sleep problems, only nightmare disorders were found to increase PTSD symptoms^34^. In agreement with the above, a 26% interceding effect of sleep insufficiency on the cross-link between blast-related traumatic brain injury (TBI) and PTSD was reported^35^. It has been reported that the adjusted odds for PTSD among service members screening positive for TBI compared to those without TBI, decreased from 1.61 (95%CI: 1.21-2.14) to 1.32 (95%CI 1.32: 0.99-1.77). A study on 2,420 soldiers during a 3-month period after deployment to Afghanistan battlefield indicated that sleep continuity disturbances could efficiently predict the link between combat exposure with PTSD. In fact, 9.5%, 6.5%, and 7.2% of participants met the criteria for PTSD, depression and GAD, respectively. An indirect association between combat exposure and PTSD was stronger in military staff with sleep duration <6 h per 24h compared to those adequate sleep quantity^36^. Clinically significant sleep disturbance assessed by the Pittsburg sleep quality index (PSQI), was reported in all 152 Australian Vietnam war veterans with PTSD and 90% of those without PTSD. Also, more serious sleep predicaments were reported among the veteran population with PTSD in the same study population^37^.

### Psychological distress and mental health problems

#### Anxiety disorders

Based on an observational study, the impaired sleep quality of military officers and soldiers from remote boundaries of China has been shown as a significant predictor for the development of anxiety symptoms. The average overall PSQI score has been reported 7 and poor sleep quality has been observed in 40.9% of the subjects. Daytime dysfunction decreased sleep efficiency and increased sleep latency were the main symptoms of the impaired sleep quality in this population^44^.

Like that, in a large military cohort, those with insomnia symptoms who slept <6h pre-deployment had increased odds of developing anxiety post-deployment than those without insomnia who retained longer sleep duration (OR=4.33, 95%CI: 2.37-7.90; OR=4.14, 95%CI: 2.09-8.22)^22^. Participants with sleep deficiency (less than 6h per 24h) were at elevated risk of a generalized anxiety disorder (GAD) (OR=9.1, 95%CI: 2.8-29.9)^25^.

Notably, according to a cross-sectional cohort study involving a large sample of U.S. Army personnel, the insomnia group was significantly more likely to have anxiety than non-insomniacs (42.7% vs. 8.0%). After controlling demographics and comorbid problems, anxiety was a statistically significant predictor of insomnia among military personnel (OR=1.93, 95%CI: 1.46-2.55)^7^.

In the same vein, the high prevalence of 82.6% was observed for insomnia disorders among military soldiers with current GAD, whereby the condition reinforced the associations between insomnia and memory/concentration problems^29^.

#### Depression

In a study on 720 military personnel in Taiwan, the average PSQI score, the average sleep latency and the average total sleep time were 7.26±3.45, 22.39±21.53 minutes, and 5.91±1.3 hours, respectively. Sleep disturbances were observed in 65.1% of subjects with a corresponding PSQI scores ≥6. In addition, a mutual relationship was found between the PSQI score and depression. Also, among 469 personnel with PSQI score above 6, 42.4% had mild to severe depression compared to 9.6% in 24 subjects with a PSQI score below 5^45^. Indeed, the pre-deployment daytime/nighttime sleep disturbances strongly contributed to the prediction of depression^24^. A report indicated that both short and long sleep duration were attributed to current major depressive disorder in veterans^23^. Deficient sleep (<6h per 24h) in individuals serving in a combat zone was a unique perspective risk factor for probable major depressive disorder (MDD) (OR=7.5; 95%CI: 2.3-25.0)^25^. Both combat and insomnia symptoms were associated with depressive symptoms^22^. Sleep problems mediated the effect of TBI on the development of depression by 41%. The adjusted odds of depressed individuals were 1.41 (95%CI: 1.11-1.80) times greater for a sailor or marine officers with TBI compared with controls^35^.

Likewise, in a sample of young men recruited in compulsory military service, the prevalence of depressive symptoms was 18% where a significant association was found between multiple related factors, e.g., stress, sleep quality, as well as circadian typology, and depressive symptoms. Along those lines, the clinically significant depressive symptoms were more prevalent among poor sleepers (PR=1.808, *p*=0.046)^46^. Based on another investigation, severe insomnia was the only strong predictor of future major depressive episodes, regardless of other symptoms such as agitation and suicidal ideation in 2,596 army recruiters^47^.

In addition to this, a study of the relationships between insomnia and depression symptoms across time periods in a sample of combat veterans also showed that insomnia at 4 months’ post-deployment time-point was a strong predictor of depression at 12 months follow-up^27^. In a cohort study including 110 active-duty service members, 70% of subjects exhibited mental disorders like depression, pain, PTSD, and mild TBI. Among military personnel with comorbid OSAHS and insomnia, 71.4% were meeting the criteria for depression^28^. A higher rate of depression was observed in insomniacs rather than controls (42.4% vs. 5.9%)^7^. Conversely, people with depression were found more likely to report insomnia (OR=2.89, 95%CI: 2.17-3.85)^7^. The comorbidity of insomnia and major depressive episode (MDE) was reported in a sample of U.S. Army soldiers and subjects with current MDE had a high prevalence of insomnia (85.0%). Given that, the MDE status influenced insomnia and cognitive insufficiencies^29^. Further to that, the prevalence of depression among 438 army soldiers was 9.6% in a study designed by Hansen et al. (2018)^31^ whereby a mutual correlation was reported between sleep problems assessed in terms of insomnia and depressive symptoms.

#### Summary

According to the present literature review, sleep predicaments seem to be notably prevalent among military members potentially owing to several psychological and physical stressors, which may likewise play a role in the pathophysiology of various mental health disorders. Sleep disturbances are considered as precipitating and perpetuating factors affecting psychiatric disorders and in turn, psychiatric disorders can reciprocally exacerbate sleep disturbances. Results of included studies demonstrate that different sleep complaints like insomnia symptoms, nightmares and problems with quantity or quality of sleep predispose military subjects to more likely experience symptoms of PTSD, depression and anxiety. A mediating role of sleep problems for the relationship between combat exposure and PTSD has also been well articulated in the literature.

Daytime and nighttime sleep complaints are shown to strongly predict PTSD and depression even after 2 years of deployment. Long and short sleep duration are also regarded as an important marker for mental disorders and risky behaviors.

Given the above, a compelling body of evidence substantiates the importance of timely screening and awareness of sleep complaints among active-duty service members and veterans. Addressing the specific sleep disturbances prior to deployment may be helpful in preventing mental health issues in this population. Moreover, as suggested by Hunt et al. (2016)^39^ since the disclosure of mental health disorders and psychological ailments in military staff is inhibited by stigmatizing beliefs, scrutinizing possible sleep disturbances needs to gain momentum.

As such, it seems essential to identify the most common mental health problems and their significant predictors such as sleep disorders within the military population to design any appropriate interventions. Future research works need to evaluate the impact of pertinent interventions to improve sleep and subsequently enhance mental resilience in this target group.

Concerning the limitations of the present review, it should be noted that included studies used various definitions and measurement tools to identify sleep disturbances. As such, it was hardly possible to perform a meta-analysis and represent the pooled predictor effects of certain sleep disturbances on mental disorders. Most of the studies monitored sleep and mental disorders using only self-report questionnaires. It is evident that objective screening measures provide more accurate and reliable data. In addition, a large number of reviewed documents had cross-sectional nature hence, identifying any causal relationship between sleep disturbances and mental health issues and generalization of the findings to a larger population were hardly feasible.

## CONCLUSION

The existing body of evidence on the links between sleep predicaments and mental health status in military staff is not vast. Findings have demonstrated the predictive role of sleep disturbances in the development of PTSD, depression and anxiety among military personnel. Given the impact of sleep disturbances on antecedent and post-deployment mental disorders among military staff, identifying any possible sleep-related issues needs further clinical attention in military medicine. Future research attempts need to examine possible predictive, preventive and personalized diagnostic and therapeutic measures in military personnel. Sleep hygiene and psychoeducation are possibly among the key substrates in the practice of sleep medicine in a military context.

## References

[1] Acheson DT, Kwan B, Maihofer AX, Risbrough VB, Nievergelt CM, Clark JW (2019). Sleep disturbance at pre-deployment is a significant predictor of
post-deployment reexperiencing symptoms. Eur J Psychotraumatol.

[2] Bliese PD, Wright KM, Adler AB, Thomas JL, Hoge CW (2007). Timing of postcombat mental health assessments. Psychol Serv.

[3] Hoge CW, McGurk D, Thomas JL, Cox AL, Engel CC, Castro CA (2008). Mild traumatic brain injury in US soldiers returning from
Iraq. N Engl J Med.

[4] Khazaie H, Nasouri M, Ghadami MR (2016). Prazosin for trauma nightmares and sleep disturbances in combat
veterans with post-traumatic stress disorder. Iran J Psychiatry Behav Sci.

[5] Raskind MA, Peskind ER, Hoff DJ, Hart KL, Holmes HA, Warren D (2007). A parallel group placebo controlled study of prazosin for trauma
nightmares and sleep disturbance in combat veterans with post-traumatic
stress disorder. Biol Psychiatry.

[6] Klingaman EA, Brownlow JA, Boland EM, Mosti C, Gehrman PR (2018). Prevalence, predictors and correlates of insomnia in US army
soldiers. J Sleep Res.

[7] Taylor DJ, Pruiksma KE, Hale WJ, Kelly K, Maurer D, Peterson AL (2016). Prevalence, correlates, and predictors of insomnia in the US Army
prior to deployment. Sleep.

[8] Kong FY, Li Q, Liu SX (2011). Poor sleep quality predicts decreased cognitive function
independently of chronic mountain sickness score in young soldiers with
polycythemia stationed in Tibet. High Alt Med Biol.

[9] Van Dongen H, Maislin G, Mullington JM, Dinges DF (2003). The cumulative cost of additional wakefulness: dose-response
effects on neurobehavioral functions and sleep physiology from chronic sleep
restriction and total sleep deprivation. Sleep.

[10] Lentino CV, Purvis DL, Murphy KJ, Deuster PA (2013). Sleep as a component of the performance triad: the importance of
sleep in a military population. US Army Med Dep J.

[11] Miller MA, Cappuccio FP (2007). Inflammation, sleep, obesity and cardiovascular
disease. Curr Vasc Pharmacol.

[12] Mysliwiec V, McGraw L, Pierce R, Smith P, Trapp B, Roth BJ (2013). Sleep disorders and associated medical comorbidities in active
duty military personnel. Sleep.

[13] Boscarino JA (2006). Posttraumatic stress disorder and mortality among US Army
veterans 30 years after military service. Ann Epidemiol.

[14] O’Toole BI, Catts SV (2008). Trauma, PTSD, and physical health: an epidemiological study of
Australian Vietnam veterans. J Psychosom Res.

[15] Cohen BE, Gima K, Bertenthal D, Kim S, Marmar CR, Seal KH (2010). Mental health diagnoses and utilization of VA non-mental health
medical services among returning Iraq and Afghanistan
veterans. J Gen Intern Med.

[16] Dobie DJ, Kivlahan DR, Maynard C, Bush KR, Davis TM, Bradley KA (2004). Posttraumatic stress disorder in female veterans: association
with self-reported health problems and functional impairment. Arch Intern Med.

[17] Killgore WD, Kahn-Greene ET, Lipizzi EL, Newman RA, Kamimori GH, Balkin TJ (2008). Sleep deprivation reduces perceived emotional intelligence and
constructive thinking skills. Sleep med.

[18] Wickwire EM, Shaya FT, Scharf SM (2016). Health economics of insomnia treatments: the return on investment
for a good night’s sleep. Sleep Med Rev.

[19] Benca RM, Obermeyer WH, Thisted RA, Gillin JC (1992). Sleep and psychiatric disorders: a meta-analysis. Arch Gen Psychiatry.

[20] Seelig AD, Jacobson IG, Smith B, Hooper TI, Boyko EJ, Gackstetter GD (2010). Sleep patterns before, during, and after deployment to Iraq and
Afghanistan. Sleep.

[21] Luxton DD, Greenburg D, Ryan J, Niven A, Wheeler G, Mysliwiec V (2011). Prevalence and impact of short sleep duration in redeployed OIF
soldiers. Sleep.

[22] Gehrman P, Seelig AD, Jacobson IG, Boyko EJ, Hooper TI, Gackstetter GD (2013). Predeployment sleep duration and insomnia symptoms as risk
factors for new-onset mental health disorders following military
deployment. Sleep.

[23] Swinkels CM, Ulmer CS, Beckham JC, Buse N, Calhoun PS, VA Mid-Atlantic MIRECC Registry Workgroup (2013). The association of sleep duration, mental health, and health risk
behaviors among US Afghanistan/Iraq era veterans. Sleep.

[24] Koffel E, Polusny MA, Arbisi PA, Erbes CR (2013). Pre-deployment daytime and nighttime sleep complaints as
predictors of post-deployment PTSD and depression in National Guard
troops. J Anxiety Disord.

[25] Taylor MK, Hilton SM, Campbell JS, Beckerley SE, Shobe KK, Drummond SP (2014). Prevalence and mental health correlates of sleep disruption among
military members serving in a combat zone. Mil Med.

[26] McLay RN, Klam WP, Volkert SL (2010). Insomnia is the most commonly reported symptom and predicts other
symptoms of post-traumatic stress disorder in US service members returning
from military deployments. Mil Med.

[27] Wright KM, Britt TW, Bliese PD, Adler AB, Picchioni D, Moore D (2011). Insomnia as predictor versus outcome of PTSD and depression among
Iraq combat veterans. J Clin Psychol.

[28] Mysliwiec V, Gill J, Lee H, Baxter T, Pierce R, Barr TL (2013). Sleep disorders in US military personnel: a high rate of comorbid
insomnia and obstructive sleep apnea. Chest.

[29] Brownlow JA, Klingaman EA, Boland EM, Brewster GS, Gehrman PR (2017). Psychiatric disorders moderate the relationship between insomnia
and cognitive problems in military soldiers. J Affect Disord.

[30] Wang HE, Campbell-Sills L, Kessler RC, Sun X, Heeringa SG, Nock MK (2019). Pre-deployment insomnia is associated with post-deployment
post-traumatic stress disorder and suicidal ideation in US Army
soldiers. Sleep.

[31] Hansen LP, Kinskey C, Koffel E, Polusny M, Ferguson J, Schmer-Galunder S (2018). Sleep patterns and problems among Army National Guard
soldiers. Mil Med.

[32] Van Liempt S (2012). Sleep disturbances and PTSD: a perpetual circle?. Eur J Psychotraumatol.

[33] Van Liempt S, Van Zuiden M, Westenberg H, Super A, Vermetten E (2013). Impact of impaired sleep on the development of PTSD symptoms in
combat veterans: a prospective longitudinal cohort study. Depress Anxiety.

[34] Steele M, Germain A, Campbell JS (2017). Mediation and moderation of the relationship between combat
experiences and post-traumatic stress symptoms in active duty military
personnel. Mil Med.

[35] Macera CA, Aralis HJ, Rauh MJ, MacGregor AJ (2013). Do sleep problems mediate the relationship between traumatic
brain injury and development of mental health symptoms after
deployment?. Sleep.

[36] Osgood JM, Finan PH, Hinman SJ, So CJ, Quartana PJ (2019). Combat exposure, post-traumatic stress symptoms, and
health-related behaviors: the role of sleep continuity and
duration. Sleep.

[37] Lewis V, Creamer M, Failla S (2009). Is poor sleep in veterans a function of post-traumatic stress
disorder?. Mil Med.

[38] Hughes JM, Ulmer CS, Hastings SN, Gierisch JM, Howard MO (2018). Sleep, resilience, and psychological distress in United States
military Veterans. Mil Psychol.

[39] Hunt E, Greenberg N, Jones N (2016). Poor sleep after military deployment: associations with mental
health difficulties. Occup Med.

[40] Kim TK, Lee HC, Lee SG, Han KT, Park EC (2016). The combined effect of sleep duration and quality on mental
health among Republic of Korea Armed Forces. Mil Med.

[41] Hougsnæs S, Bøe HJ, Dahl AA, Reichelt JG (2017). Norwegian male military veterans show low levels of mental health
problems four years after deployment in Afghanistan. Nord J Psychiatry.

[42] Soreca I, Tighe CA, Bramoweth AD (2019). The intersection of sleep apnea and severe mental illness in
veterans. Psychosomatics.

[43] Ulmer CS, Van Voorhees E, Germain AE, Voils CI, Beckham JC, VA Mid-Atlantic Mental Illness Research Education and Clinical
Center Registry Workgroup (2015). A comparison of sleep difficulties among Iraq/Afghanistan theater
veterans with and without mental health diagnoses. J Clin Sleep Med.

[44] Wang Z, Chen B, Li W, Xie F, Loke AY, Shu Q (2020). Sleep quality and its impacts on quality of life among military
personnel in remote frontier areas and extreme cold
environments. Health Qual Life Outcomes.

[45] Chou HW, Tzeng WC, Chou YC, Yeh HW, Chang HA, Kao YC (2016). Stress, sleep and depressive symptoms in active duty military
personnel. Am J Med Sci.

[46] Tonon AC, Carissimi A, Schimitt RL, Lima LS, Pereira FS, Hidalgo MP (2020). How do stress, sleep quality, and chronotype associate with
clinically significant depressive symptoms? A study of young male military
recruits in compulsory service. Braz J Psychiatry.

[47] Hom MA, Lim IC, Stanley IH, Chiurliza B, Podlogar MC, Michaels MS (2016). Insomnia brings soldiers into mental health treatment, predicts
treatment engagement, and outperforms other suicide-related symptoms as a
predictor of major depressive episodes. J Psychiatr Res.

